# Coach abusive supervision and athlete engagement: a moderated mediation model

**DOI:** 10.3389/fpsyg.2025.1494077

**Published:** 2025-04-17

**Authors:** Yuan Zeng, Junjie Yin

**Affiliations:** ^1^Department of Physical Education, Civil Aviation Flight University of China, Deyang, Sichuan, China; ^2^School of Economics and Management, Civil Aviation Flight University of China, Deyang, Sichuan, China

**Keywords:** abuse supervision, psychological empowerment, achievement motivation, athlete engagement, sports training

## Abstract

**Introduction:**

Abusive supervision remains a prominent research focus in negative organizational behavior, yet existing findings remain inconsistent. Grounded in self-determination theory, this study examines the complex relationship between coach abusive supervision and athlete engagement, while investigating the mediating role of psychological empowerment and the moderating effect of athlete achievement motivation.

**Methods:**

Using survey data from 152 athletes across Chengdu, Deyang, Mianyang, Yibin, and Zigong, we conducted hierarchical regression analyses to test: (1) the curvilinear association between abusive supervision and athlete engagement, (2) the instantaneous mediating effect of psychological empowerment, and (3) the boundary condition imposed by achievement motivation.

**Results:**

Key findings revealed: (1) An inverted U-shaped relationship between coach abusive supervision and athlete engagement; (2) Psychological empowerment mediated this relationship, with abusive supervision and empowerment also exhibiting an inverted U-shaped pattern; (3) Athlete achievement motivation significantly moderated the curvilinear relationship between abusive supervision and engagement.

**Discussion:**

This study elucidates the nonlinear transmission mechanism and psychological contingencies underlying athletes’ training engagement. The results offer theoretical contributions to sports organizational behavior literature while providing practical insights for coaches and sports administrators to optimize intervention strategies.

## 1 Introduction

With the rise of positive psychology, people have begun to pay attention to the positive psychological experience of athletes in sports, and athlete engagement has attracted the attention of more and more experts and scholars. Athlete engagement is manifested as the athlete’s mental state during sports training and exercise, and is a lasting and positive emotional cognition. In team sports, athlete engagement determines the efficiency of athletes’ technical and tactical training engagement and competitive sports performance, affects the team’s competitive style and toughness, and is even a decisive factor in winning the game (Zhang, 2012). As an important indicator of the positive aspects of athletes’ psychology, athlete engagement reflects the highly involved state of athletes’ cognition, emotion and experience. It plays a positive role in promoting the development and maturity of athletes and maximizing the overall level of individuals and teams (Ye et al., 2016). How to maintain or improve athlete engagement level has become a problem that both coaches and athletes need to pay attention to.

Existing literature on the antecedent factors of sports investment mostly focuses on the analysis of causes at the individual level, such as the “coach-athlete” relationship (Jowett et al., 2007), psychological toughness (Gucciardi et al., 2008), and coach behavior (Curran et al., 2014; Gao et al., 2021) team cohesion (Hodge et al., 2008) etc., while ignoring the interactive influence of external environment and individual psychological factors on one’s own behavior. From the perspective of self-determination theory, athlete engagement behavior is the result of the interaction between athletes’ self-awareness and external stimuli (Deci and Ryan, 2000). Influenced by the culture of superiority and inferiority, Chinese people have a greater concept of power distance. Athletes tend to obey the coaches’ orders, which provides a “soil” for coaches to carry out abusive behaviors. Abusive supervision in sports training occurs frequently. Abusive behaviors such as publicly criticizing or ridiculing athletes can send a message of inadequacy and lower performance to athletes and subject them to greater experiences and perceptions of stress. In actual training scenarios, athletes will mine key information from the coach’s behavior and integrate it into their personal subjective perception of the training experience. Athletes’ different psychological states will interpret the coach’s abusive behavior differently, thereby affecting their sports investment. Decentering capacity, which refers to the ability to detach from immediate emotional experiences, plays a critical role in athletes’ psychological resilience, potentially influencing their engagement levels under varying supervisory conditions (Diotaiuti et al., 2023). Based on the above discussion, this study explores the mediating mechanism of psychological empowerment between coaches’ abusive supervision and athletes’ sports engagement based on self-determination theory. In addition, achievement motivation theory shows that the pursuit of excellence is an individual’s intrinsic motivation in career growth and development (Natalia et al., 2021). Athletes’ different levels of achievement motivation mean that they have different intrinsic motivations in team training and sports competitions, and therefore their perceptions and reactions to abusive supervision by coaches are also different. Therefore, this study further examines the moderating role of achievement motivation between abusive supervision by coaches and athletes engagement in sports.

Through the above exploration, this study attempts to promote a more comprehensive and in-depth understanding of the effectiveness of abusive supervision in theory and practice. On the basis of clarifying the impact of coaches’ abusive supervision on athlete engagement in sports, this study attempts to identify the differences in athletes’ achievement motivations in the above paths. The research results can provide theoretical support for athletes to improve their athlete engagement levels, and at the same time point out the direction for sports managers to effectively intervene in sports engagement.

## 2 Theoretical explanation and hypothesis

### 2.1 Abusive supervision and athlete engagement

In this study, the coach’s abusive supervision refers to the persistent hostility behavior carried out by the coach when the athlete perceives it (Tepper, 2000), such as ridiculing the athlete, publicly criticizing, belittling and questioning the athlete’s ability, etc. Relevant studies have pointed out that abusive supervision, as a stress source, can stimulate different coping behaviors in individuals.

According to self-determination theory, differences in incentive levels can trigger different levels of individual psychological activity. Too low or too high an incentive level is not conducive to personal performance. Only at a moderate level of activity can an individual’s external perception and psychological state achieve the best match. As an important source of activation in training venues, moderate abuse supervision is conducive to athletes optimizing their mental state and increasing their enthusiasm for training (Bhattacharjee and Sarkar, 2024). At this time, the main purpose of abusive supervision is negative feedback and timely correction of errors, that is, the coach releases information through a negative feedback loop to allow athletes to recognize errors that should not occur in training, or to motivate athletes to work hard to improve skills and abilities. Therefore, moderately abusive supervision is intended to express dissatisfaction with the athlete’s attitude, ability, or performance level, and to hope that the athlete will make behavioral changes (Velez and Neves, 2016). Athletes will adjust their self-perception and behavior by interpreting the external environment, thereby viewing the coach’s hostile behavior as motivation to improve their athletic performance. In this case, the coach’s abusive behavior is more similar to an external supervision method, which can motivate athletes to produce proactive behaviors. Podsakoff and Farh (1989) found that compared with positive feedback, individuals will adjust their goal height upward after receiving negative feedback, thereby stimulating their own motivation and work engagement level. Wee (Wee et al., 2017) also pointed out that individuals usually do not choose confrontational behavior to deal with abuse from their superiors, but work harder to improve their self-worth in order to increase their superiors’ reliance on their power and break the abuse spiral in the workplace. Based on this, we believe that when faced with the pressure brought by moderately abusive supervision, athletes will increase their sports engagement based on the perceived deviation, cater to the coach’s expectations for improving their abilities and performance, and alleviate negative pressure experiences.

When the level of abusive supervision is low, the level of activation experienced by athletes is also relatively low, which in turn cannot effectively stimulate individual sports enthusiasm and prevent athletes from having a good psychological state to face training bottlenecks. When the level of abusive supervision is high, it will seriously affect athletes’ cognitive and emotional adjustment abilities, resulting in a decrease in individual security (Lee et al., 2019), making athletes think that the coach does not recognize their efforts and changes in sports, which may arouse their natural resistance and revenge. Affected by the traditional concept of superiority and inferiority, athletes will use self-control to suppress the urge to retaliate against the coach’s abusive behavior. However, if this kind of abusive behavior exceeds a certain limit, it will cause athletes to suffer intense ego-depletion, causing problems such as reduced efficiency and negative movement during training and competition, and even lead to counterproductive behaviors such as retaliation against the team.

Based on the above mentioned, hypothesis H1 is proposed: Coach’s abusive supervision has an inverted U-shaped impact on athletes engagement in sports.

### 2.2 The mediating role of psychological empowerment

Psychological empowerment is an athlete’s perception of the training environment from four dimensions: sport significance, self-efficacy, self-determination and influence (Zheng and Liu, 2016). This “empowered” psychological perception is greatly affected by coaches, mainly because the coach’s supervision style works by activating cognitive, affective, and interpersonal mechanisms. Recent research highlights the importance of psychological constructs, such as decentering, in mediating the relationship between external stressors and athlete engagement, providing further evidence of the nuanced interactions at play (Diotaiuti et al., 2023). Because of the coach’s ability to influence an athlete’s resources and career development, his or her behavior has always been an athlete’s most important source of information. When athletes are abused by their coaches, their perceptions of psychological empowerment change, causing them to make different choices about their behavior. When the coach’s abuse is at an appropriate level, athletes will regard appropriate criticism and correction as an external motivation. This appropriate external driving force will enhance the athlete’s training autonomy and self-efficacy (Natalio et al., 2018), thereby enhancing their psychological empowerment perception. However, when the coach’s abuse level is too high, athletes will feel that their efforts and efforts have been ignored or belittled, doubt their athletic ability and the significance of training, and even have feelings of escape and resistance. This negative cognitive and emotional experience will lead to negative sports attitudes of athletes and reduce the perception of psychological empowerment.

Based on this, hypothesis H2 is proposed: Abusive supervision has an inverted U-shaped impact on psychological empowerment.

Self-determination theory emphasizes the importance of satisfying individual needs in promoting sports engagement, and psychological empowerment is regarded as one of the important manifestations of satisfied individual needs. For athletes, psychological empowerment means they feel a sense of autonomy, competence, and relatedness in their sport (Wang et al., 2022). When athletes have higher levels of psychological empowerment, they are more likely to have positive attitudes and emotions toward athletic training and competition. They have more trust in their abilities and potential, have sufficient self-efficacy to complete difficult training tasks, are willing to assume more responsibilities and obligations, and are more willing to engage time and energy in sports training (Zhong et al., 2011). Athletes with high levels of psychological empowerment are also more likely to proactively seek challenges and opportunities to improve their skills and performance and work toward achieving their goals. On the contrary, athletes with low levels of psychological empowerment lack the confidence to complete sports training, are skeptical about their abilities and potential, feel that their efforts have minimal impact on achieving goals, and even feel frustrated and anxious, and are less willing to put more effort into their goals or devote their energy to sports training. In addition, athletes with low levels of psychological empowerment may not fully perceive the meaning and value of sports training. They may feel that training is an externally imposed task rather than motivated by love and interest in the sport itself. This external motivation may lead to athletes lacking intrinsic drive, enthusiasm and initiative in training and competition, and being unwilling to give their best. In summary, this study proposes the following hypotheses.

*H3*: Psychological empowerment positively affects athletes’ sports engagement.

*H4*: Psychological empowerment has a mediating effect on the relationship between abusive supervision and athlete engagement.

### 2.3 The moderating effect of athletes’ achievement motivation

Self-determination theory emphasizes that the implementation of people’s behavior is the result of the combined effect of internal and external motivations. Achievement motivation is the drive an individual exhibits when completing tasks and achieving goals. It stems from an individual’s pursuit of self-growth and development, as well as their confidence and expectations in their ability to successfully complete tasks and goals. When individuals have strong achievement motivation, they will usually work harder, overcome difficulties, and pursue excellence to achieve their goals (Li et al., 2022; Bektas and Sohrabifard, 2013). Research shows that achievement motivation is an important invisible hand that motivates individuals to actively acquire resources and seek meaning and value. It determines the intensity of individual implementation behaviors and has the effect of “self-improvement leads to personal changes” (Hakanen et al., 2017). Individuals with different levels of achievement motivation have different expectations of success, which makes them perceive and react differently to leadership feedback behaviors (Petrou et al., 2012; Wang et al., 2014). When individuals have high achievement motivation, they are more likely to devote more time and energy to completing tasks and strive to exceed their abilities and levels. This effort and commitment leads to better performance and results. It can be inferred that achievement motivation may have a contingency effect on the relationship between abusive supervision by coaches and athlete engagement in sports.

First of all, if athletes have high achievement motivation, they will have a stronger desire for success, hoping to achieve success through their own efforts and performance, and to be recognized and appreciated by those around them. In order to achieve success, they also pay more attention to how to obtain the resources around them, especially actively seeking help from coaches. Secondly, athletes with high achievement motivation pay more attention to their own growth and development, and have a stronger need to improve their sports skills (Chen et al., 2020). The targeted guidance, support and feedback provided by coaches in abusive supervision can greatly improve athletes’ performance. Sense of competence. Finally, athletes with high achievement motivation have stronger needs for relational resources, and coaches’ daily close interaction with them is conducive to satisfying athletes’ belonging needs (Li, 2010). Secondly, athletes with high achievement motivation pay more attention to their own growth and development, and they have a stronger need to improve their athletic ability. The sports guidance, technical support and information feedback provided by coaches during abusive supervision have greatly improved the athletes’ sense of competence. This means athletes feel they have the help they need and feel more confident and motivated to confront challenges. Finally, athletes with high achievement motivation usually have a stronger need for relational resources. They not only focus on the results of the game, but also value relationships with coaches, teammates, etc. They want to develop a deep trusting relationship with their coach while receiving additional support and guidance. Coaches’ daily interactions with them help satisfy athletes’ belonging needs. Not only does this interaction help build closer relationships, it also makes athletes feel seen and included, thereby increasing their sense of belonging and engagement.

In summary, athletes with high achievement motivation have high expectations for their own performance and are willing to work hard to improve their abilities and achieve success. Faced with abusive supervision, they may become more focused on how to learn, grow, and improve themselves from it. This mentality makes them more willing to seize learning opportunities and regard abusive supervision as a challenge and motivation, thereby enhancing their recognition of the meaning and value of sports; On the contrary, athletes with low achievement motivation have lower requirements for their own performance and lack the desire for self-improvement. They have smaller needs for skill learning, motor feedback, etc., and therefore may be relatively less responsive to abusive supervision. Rather than viewing abusive supervision as a challenge or motivation, they are more likely to view it as a negative factor, thereby reducing their engagement in the sport. Therefore, the hypothesis is proposed:

*Hypothesis 5*: Athletes’ achievement motivation mediates the relationship between coaches’ abusive supervision and athlete engagement, that is, when athletes’ achievement motivation is higher, the inverted U-shaped relationship between coaches’ abusive supervision and athlete engagement in sports is stronger.

**Figure 1:**
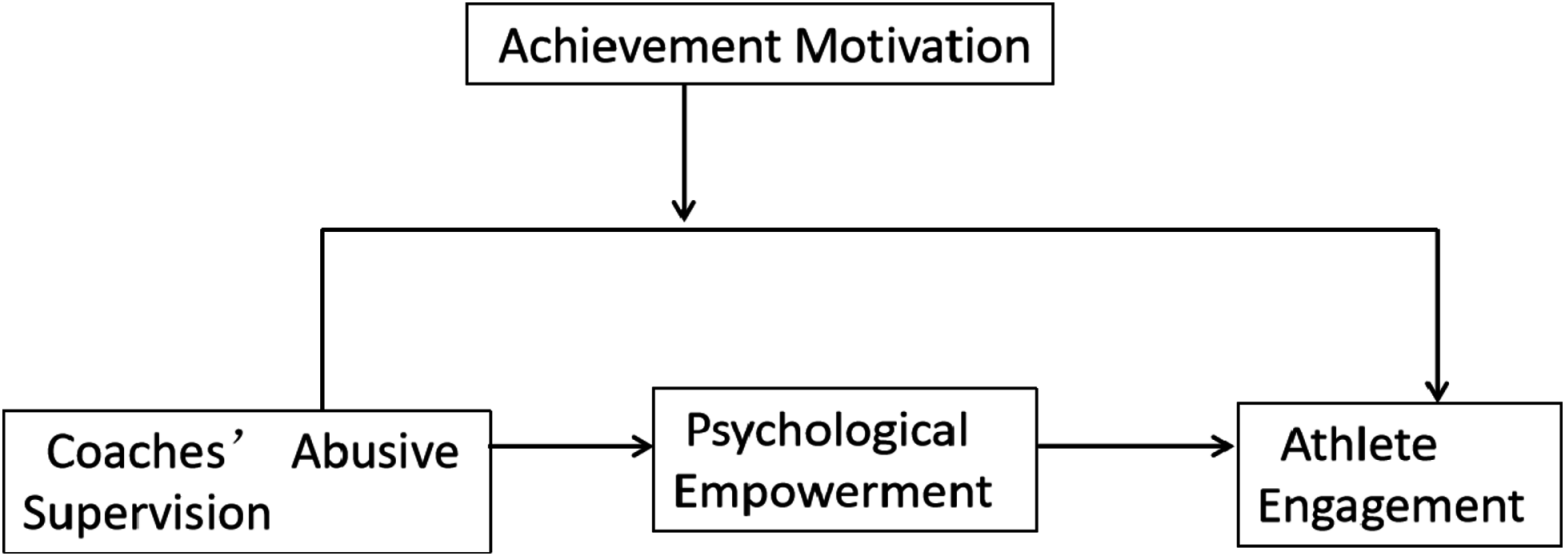
Theoretical model framework.

The research model of this study is shown in Figure 1.

## 3 Research design

### 3.1 Research sample and data collection

This study selected athletes from Chengdu, Deyang, Mianyang and other places who are engaged in swimming, gymnastics, diving, football, basketball, martial arts and other sports to participate in this research investigation. The entire investigation process lasted 3 months (March 2023–May 2023). First, contact the athletes being tested to inform them of the content, purpose, data collection process and other information of this study, and confirm their willingness to participate. In the end, a total of 152 athletes agreed to participate in this survey. Secondly, a WeChat group was established for athletes who confirmed to participate in the survey to facilitate subsequent questionnaire distribution and collection. Finally, the specific questionnaire distribution and collection work was carried out in two stages. The first research phase mainly collects information on athletes’ individual characteristics (such as age, gender, sports skill level, etc.). The second phase of data collection will be carried out 1 week later. Athletes need to fill in the same set of questionnaires every day for a total of 7 training days. The questionnaire consists of two parts. The first part includes the abusive supervision, achievement motivation and psychological empowerment scales, and the second part is the sports engagement scale.

At 13: 30 on each training day, the athletes are required to complete the first part of the questionnaire. This time point is chosen because the athletes have trained all morning and have the opportunity to feel the abuse from the coach and perceive their own achievement motivation and psychological empowerment. At 18: 30 on each training day, athletes must complete the second part of the questionnaire. Filling out the questionnaire twice every day ensures the causal relationship between the independent variables and the dependent variables to a certain extent. In the end, a total of 1,064 questionnaires were distributed. Because some athletes did not complete the questionnaire for all 7 days, and some data were missing, after eliminating invalid questionnaires, this questionnaire survey finally obtained 808 valid samples, and the valid sample ratio was 75.94%.

The total 808 valid samples were analyzed through SPSS. The results showed that: 64 people were female (42.10%) and 88 were male (57.89%); among them, 45 (29.60%) were between 10 and 15 years old, 71 (46.71%) were between 16 and 18 years old, and 36 (23.68%) were between 19 and 24 years old; 11 master athletes (7.237%), 56 first-level athletes (36.84%) and 85 s-level athletes (55.92%).

### 3.2 Variable measurement

In order to ensure the validity and reliability of the measurement, the sources of the scales are mature scales published in top international and domestic journals and have been used in China. Measurement uses a 7-point Likert scale scoring method.

Abusive supervision. The scale is based on the scale compiled by Aryee et al. (2008) and was revised based on athletes’ feelings when they face abusive supervision. It has a total of 10 items. A sample item is “My coach often says that I am not good enough.” The Cronbach’s *α* coefficient of this scale is 0.861.

Psychological empowerment. The 12-item psychological empowerment scale translated by scholar Li et al. (2006) was adopted. The sample item was “The training I did was very meaningful to me.” The Cronbach’ s *α* coefficient of this scale is 0.948.

Achievement motivation. The scale draws on the measurement method of Ye et al. (1992) and includes a total of 6 items. A sample item is “I like novel and difficult tasks, and I even do not hesitate to take risks.” The Cronbach’s α coefficient of this scale is 0.847.

Athlete engagement in the sports. The scale adopts the exercise involvement scale compiled by Lonsdale et al. (2007). The scale contains 16 items in the four dimensions of confidence, energy, dedication and enthusiasm. The sample item is “I am very focused during training and competitions.” The Cronbach’s α coefficient of this scale is 0.824.

## 4 Results and analysis

### 4.1 Common method bias and discriminant validity test

The study uses questionnaires filled in at different times, but the measurement of abusive supervision comes from athletes’ perception of coach’s behavior, and psychological empowerment, achievement motivation and athlete engagement are all individual psychological variables. The questionnaire data are filled in in the form of self-report, which may have the problem of common method bias. The Harman single factor test showed that four factors were separated out without rotation, and the factors explained a total of 74.64% of the variation. The variation explained by the first principal component factor was 28.76%, which was lower than 50%, indicating that there is no obvious common method bias problem in the recovered data.

The study used Amos to conduct confirmatory factor analysis on the variables in the model (abusive supervision, achievement motivation, psychological empowerment and athlete engagement) to test the discriminant validity between variables. The results in Table 1 show that the fit index of the four-factor model is better than that of the competing models. Therefore, the four-factor model has better discriminant validity.

**Table 1:** Model comparison of confirmatory factor analysis.

Model	Factor	*χ* ^2^	df	*χ*^2^/df	CFI	IFI	NNFI	RMSEA
Quartet	AS, PE, AM, AE	626.175	253	2.475	0.956	0.934	0.972	0.054
Three-factor	AS+PE, AM, AE	1699.328	256	6.638	0.764	0.791	0.682	0.157
Two-factor	AS+PE + AM, AE	2345.162	262	8.951	0.615	0.705	0.612	0.214
One-factor	AS+PE + AM+AE	3206.016	264	12.144	0.473	0.462	0.469	0.233

AS stands for abusive supervision, PE stands for psychological empowerment, AM stands for achievement motivation, and AE stands for athlete engagement.

### 4.2 Descriptive statistics and correlation analysis results

Table 1 lists the means, standard deviations, and correlation matrices of each variable. As can be seen from Table 2, there is a significant positive correlation between psychological empowerment and athlete engagement (*r* = 0.104, *p* < 0.01), and Hypothesis 3 has been preliminarily verified. Abusive supervision was not related to athletic engagement, providing a basis for subsequent testing of the hypothesis. Through data analysis, it can also be found that the correlation coefficients of each variable are less than 0.7, indicating that there is no potential collinearity problem.

**Table 2:** The mean, standard deviation and correlation coefficient of each variable.

Variable	1	2	3	4	Mean value	Standard deviation
1. AS	—				2.756	0.825
2. PE	0.183^*^	—			3.482	0.716
3. AM	−0.178^**^	0.232^*^	—		2.835	0.860
4. AE	−0.218	0.104^**^	0.112^*^	—	3.226	0.732

* and ** indicate significant at *p* < 0.05 and *p* < 0.01 levels, respectively, (two-tailed test), the same as below.

### 4.3 Hypothesis test results

**Figure 2:**
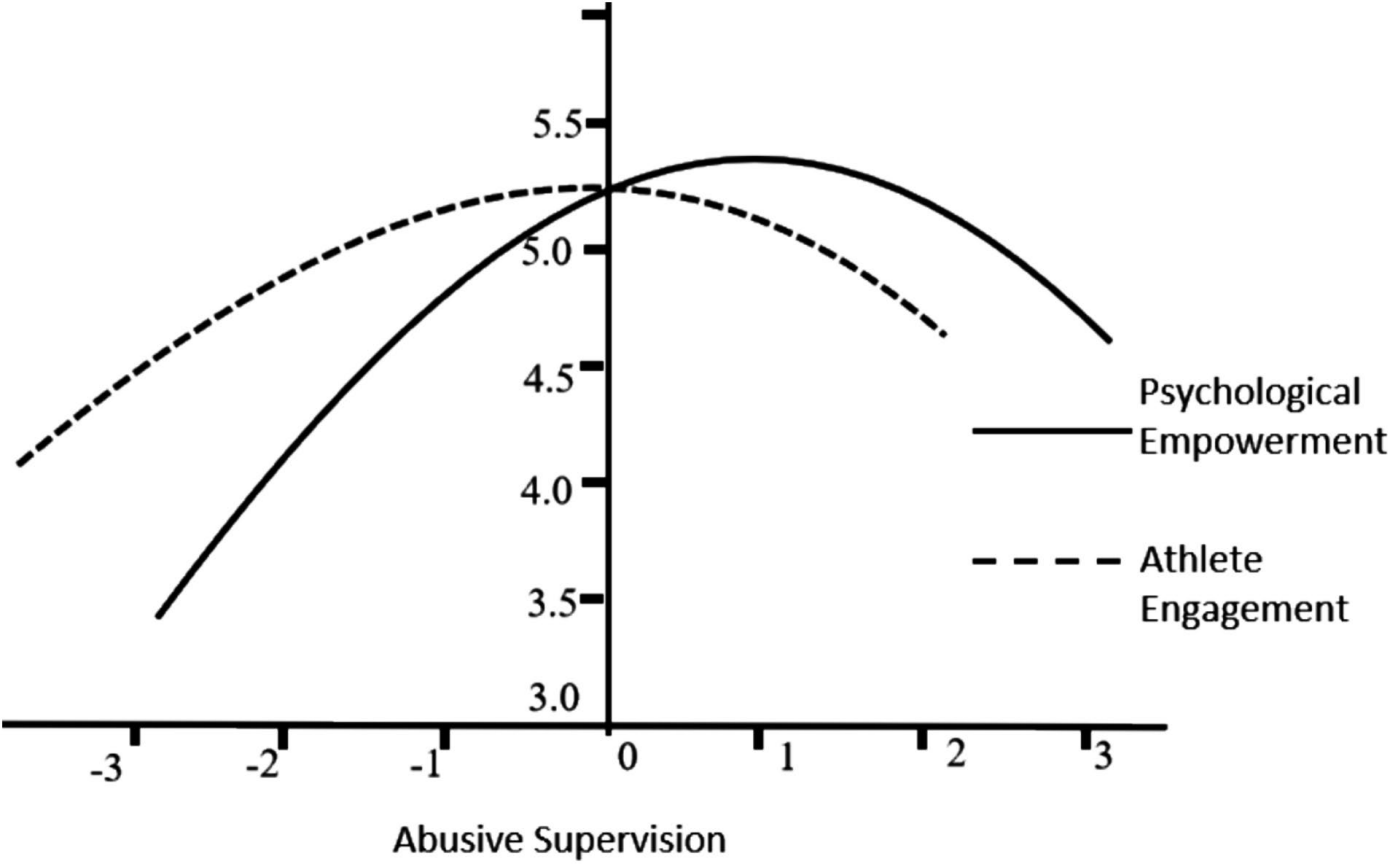
Relationship between abusive supervision and athletes’ psychological empowerment and engagement.

According to model 2 in Table 3, abusive supervision has a significant positive impact on athlete engagement in sports (*b* = 0.138, *p* < 0.05), while the quadratic term of abusive supervision has a significant negative impact on athlete engagement (*b* = −0.214, *p* < 0.01). Therefore, hypothesis 1 is confirmed. Figure 2 illustrates the inverted U-shaped relationship between abusive supervision and athlete engagement in sports. Model 3 is used to test the relationship between psychological empowerment and athlete engagement in sports. The regression coefficient of psychological empowerment is 0.431 (*p* < 0.01), and hypothesis 3 is confirmed.

**Table 3:** Multilevel regression results.

Variable	Athlete engagement				Psychological empowerment	
	Model 1	Model 2	Model 3	Model 4	Model 5	Model 6
Athlete age	−0.015	−0.013	−0.018	−0.007	−0.010	−0.009
Athlete gender	0.052	0.068	0.043	−0.023	0.032	0.012
Athlete level	−0.017	−0.021	−0.022	0.055	0.075	0.062
Time spent with the coach	0.016	0.021	0.016	0.042	0.018	0.016
Age of the coach	0.002	0.006	0.015	0.016	0.004^***^	0.007
Coach education level	0.013	0.021	−0.017	−0.002	−0.006^**^	−0.003^***^
AS	0.106^*^	0.138^*^		−0.191	0.387^**^	0.238^***^
AS squared		−0.214^**^		−0.078^**^		−0.167^*^
PE			0.431^**^			
AM				0.189		
AM × AS				0.327^**^		
AM × AS squared				0.485^*^		
Pseudo-*R*^2^		0.189	0.237	0.384	0.096	0.115

* and ** indicate significant at *p* < 0.05 and *p* < 0.01 levels, respectively, (two-tailed test). *** indicates significant at *p* < 0.001 level (two-tailed test).

Models 5 and 6 are used to examine the relationship between abusive supervision and psychological empowerment. The regression coefficient of the linear term of abusive supervision in Model 5 is 0.387 (*p* < 0.01), and the coefficient of the quadratic term of abusive supervision in Model 6 is −0.167 (*p* < 0.05). And Pseudo-*R*^2^ shows that after adding the square term of abusive supervision, the proportion of variance explained in psychological empowerment increases by 11.5%, indicating that there is an inverted U-shaped curve relationship between abusive supervision and psychological empowerment, and hypothesis 2 has been verified.

The relationship between abusive supervision, psychological empowerment and athlete engagement is shown in Figure 2. The zero slope point of the above inverted U-shaped curve is calculated according to the method of Antonakis et al. (2017). The results show that the zero slope point of psychological empowerment and athlete engagement are located at the mean distance of abusive supervision at 0.914 and −0.385 units, respectively. This shows that a moderate level of abusive supervision can positively affect athlete engagement in sports. After exceeding the zero slope point (i.e., −0.385 units on the abscissa in Figure 2), abusive supervision will negatively affect athlete engagement. Similarly, Figure 2 also reveals an inverted U-shaped relationship between abusive supervision and psychological empowerment.

**Figure 3:**
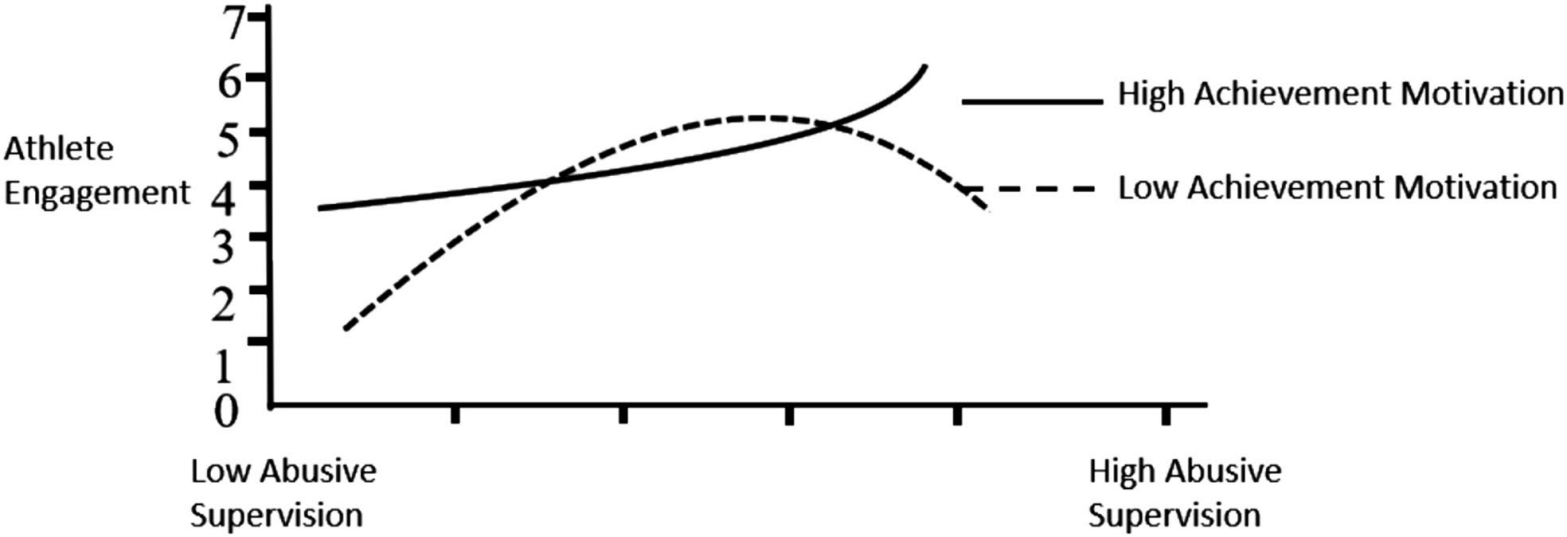
Achievement motivation moderating effect diagram.

This study calculates the instantaneous indirect effects under different values of abusive supervision based on the coefficients in Model 2 in Figure 3. The results show that when abusive supervision takes a low value (mean−1 standard deviation) and the mean, the instantaneous indirect effects are, respectively, 0.112 and 0.072, the 95% confidence intervals are [0.069, 0.227] and [0.027, 0.148] respectively, both of which do not include 0, that is, moderate abusive supervision, which has a significant mediating effect on athlete engagement in sports through psychological empowerment. When abusive management takes a high value (mean + 1 standard deviation), the instantaneous indirect effect is −0.008, and the 95% confidence interval is [−0.035, 0.029], including 0, indicating that the mediating effect is not significant at this time. Furthermore, judging from the value of the mediating effect (0.112 > 0.072 > −0.008), the mediating effect decreases with the increase of abusive supervision. Therefore, moderate abusive supervision can improve athlete engagement in sports through the indirect effect of psychological empowerment; however, when abusive supervision is excessive, it cannot improve athlete engagement through the indirect effect of psychological empowerment. H3 is supported by empirical data.

Model 4 in Figure 3 shows that the interaction term between abusive supervision and athletes’ achievement motivation positively affects athlete engagement (*b* = 0.327, *p* < 0.01), and the interaction term between the quadratic term of abusive supervision and athletes’ achievement motivation is positively significant. Affects exercise investment (*b* = 0.485, *p* < 0.05). Therefore, athletes’ achievement motivation plays a moderating role in the relationship between abusive supervision and athlete engagement, and athletes’ achievement motivation strengthens the inverted U-shaped relationship between the two. Hypothesis 5 is thus confirmed (as shown in Figure 3).

Based on the results of Model 3 in Table 3, this study calculated the instantaneous indirect effect of abusive supervision on athlete engagement through psychological empowerment under different values of athletes’ achievement motivation. It can be seen from Table 4 that when athletes’ achievement motivation is low (mean−1 standard deviation), the instantaneous indirect effects of abusive management at low, medium and high values are −0.014 (*p* > 0.05) and −0.053 (*p* < 0.05), −0.132 (*p* < 0.05); when athletes’ achievement motivation is high (Mean +1 standard deviation), the instantaneous indirect effects of abusive supervision at low, medium and high values are 0.117 (*p* < 0.05), 0.128 (*p* < 0.05), 0.129 (*p* < 0.05) respectively. It can be seen that under different levels of achievement motivation of athletes, there are significant differences in the instantaneous indirect effects of abusive supervision on athlete engagement in sports through psychological empowerment. Therefore, Hypothesis 5 is further verified.

**Table 4:** Mediation of moderated curvilinear.

Achievement motivation	Abusive supervision	Instantaneous indirect effect	95% Confidence intervals
Low (mean-1 standard deviation)	High (mean + 1 standard deviation)	−0.014	[−0.187, 0.040]
	Medium (mean)	−0.053^*^	[−0.231, -0.016]
	Low (mean−1 standard deviation)	−0.132^*^	[−0.365, -0.024]
High (mean+1 standard deviation)	High (mean+1 standard deviation)	0.065^*^	[0.019, 0.186]
	Medium (mean)	0.128^*^	[0.037, 0.212]
	Low (mean−1 standard deviation)	0.136^*^	[0.048, 0.236]

* indicate significant at *p* < 0.05 (two-tailed test).

## 5 Argumentation

Under my country’s unique “nation-wide system” competitive sports training and management model, coaches play a decisive role in the growth of athletes in the process of practicing the Olympic purpose of “Peace, Friendship, and Progress.” Mutual respect and trust between coaches and athletes are important factors for athletes to achieve successful performance. In the sports world, there have also been successful cases such as “Sun Haiping-Liu Xiang,” “Lang Ping-Zhu Ting,” and “Ans Botha-Wayde van Niekerk.”

However, the rigid “coach-athlete” relationship caused by abusive supervision is also a common phenomenon in sports training. This relationship will not only destroy the continuity and systematicness of sports training, but will even seriously affect the physical and mental health of athletes. If the coach does not handle the management model of the athletes well, it may cause serious deviations in their future career growth. Based on the self-determination theory, this study analyzes the impact mechanism of coaches’ abusive supervision on athlete engagement in sports, and examines the mediating role of athletes’ psychological empowerment and the moderating role of achievement motivation, thus to provide theoretical basis with a view to promoting athlete engagement levels for coaches and sports managers.

### 5.1 The impact of coaches’ abusive supervision on athlete engagement

There is an inverted U-shaped relationship between coaches’ abusive supervision and athlete engagement, that is, moderate abusive supervision can help improve athletes engagement. However, as the coach’s abusive behavior increases, the positive effect gradually decreases. When the degree of abuse exceeds the corresponding threshold, athlete engagement is suppressed.

Coaching behavior in sports training often contains the element of “establishing authority.” In order to strengthen the team’s obedience, ensure the efficient operation of the team, and avoid improper competition, coaches often show an authoritarian style and demean the abilities of athletes, which to a certain extent helps to establish their own authority. This “establishing power” element is often directed at tasks rather than individual attacks, just like “the beating of a stick produces a filial son” and “nothing is effective without beatings.” It is easier to convey a direct and powerful signal than the traditional implicit and roundabout expressions, allowing athletes to knowing the coach’s expectations, standards and bottom-line, which will help improve athlete engagement (Jones et al., 2007). However, as the degree of abuse increases, athletes’ negative emotional experience will continue to increase, and their perception of abusive supervision will also shift from sports training to individual attack, resulting in an inverted U-shaped relationship between abusive supervision and athlete engagement.

Existing research focuses on the negative effects of abusive supervision on subordinates’ attitudes and behaviors, but insufficient attention is paid to its positive effects. The research results responded to the call of relevant scholars to study the curvilinear relationship of complex variables in management (Zhang and Liu, 2018), revealed the possible positive effects of destructive leadership behaviors such as abusive supervision, and found that there is an optimum that is most conducive to athletes’ psychological empowerment and athlete engagement. The level of supervision further clarified the induction mechanism of athlete engagement in sports, deepened the exploration of the potential positive effects of abusive supervision, and also provided a new perspective for further exploration of the positive effects of abusive supervision in the future.

### 5.2 The mediating role of athletes’ psychological empowerment

Based on the self-determination theory, this study verifies the transmission effect of psychological empowerment between abusive supervision and athlete engagement in sports, and there is also an inverted U-shaped relationship between abusive supervision by coaches and athletes’ psychological empowerment. A moderate level of abusive supervision can better stimulate athletes’ psychological empowerment, thereby affecting their athlete engagement.

Through the creation of a training environment and the construction of individual psychological perceptions, abusive supervision provides an external motivation platform for athletes as a basis for exercising their own abilities and status. However, abusive supervision requires psychological empowerment to transform the atmosphere formed by the external environment into psychological factors that can be felt, so as to influence the behavior of athletes. Appropriate use of abusive supervision can be used as a source of activation in the training venue, helping to stimulate the intrinsic motivation of athletes, prompting them to overcome difficulties, surpass themselves, increase training enthusiasm, and then optimize their psychological state. However, when faced with excessive abuse by coaches, athletes feel that their expectations for the coach have not been met, and they may experience emotions such as anger and disappointment. However, due to the influence of the culture of superiority and inferiority, athletes usually do not have direct conflicts with coaches. Instead, they choose to adopt negative behaviors such as avoidance and compromise in response to the coach’s abusive behavior. This will have a great negative psychological impact on the athletes. Thus negatively affecting psychological empowerment. The findings align with recent studies on decentering capacity in athletes, suggesting that individual psychological traits significantly moderate the impact of external supervision styles on athlete engagement (Diotaiuti et al., 2023). In addition, in the field of organizational behavior, scholars usually explore psychological empowerment as individual internal motivation; while in the field of sports psychology, individual internal motivation is considered to be a key factor affecting athlete engagement. Psychological empowerment allows athletes to feel that they are part of the organization, with the ability and freedom to determine their own behavior and participate in team decisions, thereby increasing self-efficacy and perceptions of autonomy in sports training. These experiences enable athletes to engage more proactively with the organization, strive to improve athletic efficiency, and develop their potential to cope with challenges and pressures.

Since there are currently no relevant studies exploring and testing the mediating effect of psychological empowerment between coaching behavior and athlete engagement in sports, the results of this study can be regarded as a useful supplement to previous research to a certain extent. It not only helps to enrich the antecedent variables of psychological empowerment, but also helps to clarify the mechanism of abusive supervision.

### 5.3 The moderating role of athletes’ achievement motivation

The study also confirmed that athletes’ achievement motivation plays a moderating role in the inverted U-shaped relationship between abusive supervision and athlete engagement in sports. When athletes’ achievement motivation is high, the inverted U-shaped relationship between abusive supervision and athletes engagement is stronger.

As a relatively stable personal trait of athletes, achievement motivation is the internal driving force for athletes to overcome obstacles and achieve outstanding results. It affects the choice of individual goals, strategies to achieve goals, and the level of effort, and plays an important role in the growth of athletes (Schoen, 2015). Athletes with high achievement motivation have higher demands on themselves, are more likely to set challenging sports goals, and actively explore more opportunities to improve their performance. In the process of facing abusive supervision by coaches, athletes will be more proactive in catering to the coach’s expectations, actively seeking support and help from teammates, devoting time and energy to completing training goals, and pursuing excellence and success by working harder than others (Kosmidou et al., 2013). On the contrary, athletes with low achievement motivation have low self-efficacy and lack interest and intrinsic drive in sports training. In abusive supervision situations, athletes will suffer tremendous psychological pressure and pain, and even need to spend more time and energy dealing with the adverse relationship with their coaches. As a result, athletes will avoid daily team training and athlete engagement. The research results clarify the boundaries for a more accurate understanding of the differential mechanism of abusive supervision on athlete engagement. This finding has good reference value for deepening research on abusive supervision.

### 5.4 Enlightenment on the study

First of all, abusive supervision is a “double-edged sword” in terms of improving athletes’ psychological empowerment and athlete engagement in sports. Coaches need to update their management concepts. Traditional abusive supervision does not necessarily promote the improvement of athletes’ sports level. In organizing training practices, attention should be paid to controlling abusive supervision to a moderate level to achieve the best training effect. Care must be taken not only to prevent athletes from lacking sufficient incentives to challenge themselves and break through their limits, but also to prevent excessive abusive supervision from having a negative impact on the physical and mental health of athletes. Therefore, in sports training, we should pay attention to the abusive supervision behaviors that often exist under the high traditional concept of respect and inferiority, and give full play to the positive role of abusive management. Coaches must be clear about the direction of abusive supervision and effectively balance the “degree” of their own abusive behavior. When criticizing athletes, they need to focus on specific deficiencies in their training and their possible causes, rather than arbitrarily belittling the overall level of athletes, or accuse them of personality flaws. In particular, coaches must avoid certain highly harmful abusive behaviors, such as infringing on athletes’ privacy, not keeping promises, etc. These management methods will not only fail to inspire active coping behaviors in athletes, but will instead trigger deviant behaviors.

Secondly, pay attention to the psychological empowerment of athletes and provide a “stimulant” to improve the intrinsic motivation of athletes. As an important link between external resources and internal driving forces, psychological empowerment enhances athletes’ positive feelings in the process of self-growth and development, making it easier for athletes to show positive behaviors and attitudes, such as higher self-esteem, stronger self-confidence, better adaptability, etc. Therefore, measures such as emphasizing to athletes the importance of the training they are engaged in, creating a comfortable sports environment, and granting self-determination rights can improve their psychological empowerment and stimulate their sports potential, thereby making the team more cohesive.

Finally, coaches can focus on high-achievement motivated athletes and provide them with more support. In addition, training, learning, team atmosphere and other methods can be used to strengthen athletes’ understanding of achievement motivation, and encourage athletes to improve their achievement motivation levels in many aspects, so as to better play the positive role of abusive supervision.

### 5.5 Research deficiencies and prospects

Certainly, the research design of this paper still has some shortcomings. This study selected exercise input as the criterion, which is in line with the general practice of sports psychology. But objective results are perhaps the most compelling evidence. In the future, subjective and objective performance can be combined to fully verify the effect of abusive supervision by coaches. Furthermore, although the sample includes athletes from various disciplines, future studies with greater diversification could improve the generalizability of the results. At the same time, the differences in subjective initiative and self-perception caused by age and gender differences are not fully considered. Meanwhile, the study does not sufficiently detail the influence of cultural differences on the perception of abusive supervision. Follow-up research can consider relevant factors to improve the research content.

## 6 Conclusion

(1) There is an inverted U-shaped relationship between coaches’ abusive supervision and athlete engagement in sports.

(2) Psychological empowerment has a mediating effect on the relationship between abusive supervision and athletes engagement, and there is also an inverted U-shaped relationship between coaches’ abusive supervision and athletes’ psychological empowerment.

(3) Athletes’ achievement motivation plays a moderating role in the inverted U-shaped relationship between abusive supervision and athlete engagement in sports.

Research results show that moderately abusive supervision can help improve athletes’ psychological empowerment, thereby promoting their athlete engagement in sports; in abusive supervision, athletes’ achievement motivation should be focused on and cultivated, rather than blindly emphasizing dominance and submission.

## Data Availability

The raw data supporting the conclusions of this article will be made available by the authors, without undue reservation.
